# Bone Metabolism in SARS-CoV-2 Disease: Possible Osteoimmunology and Gender Implications

**DOI:** 10.1007/s12018-020-09274-3

**Published:** 2020-09-01

**Authors:** Gianmaria Salvio, Claudio Gianfelice, Francesca Firmani, Stefano Lunetti, Giancarlo Balercia, Gilberta Giacchetti

**Affiliations:** Division of Endocrinology, Department of Clinical and Molecular Sciences, Polytechnic University of Marche, Via Conca 71, Umberto I Hospital, 60126 Ancona, Italy

**Keywords:** Osteoporosis, Fracture risk, COVID-19, SARS-CoV-2, Denosumab

## Abstract

Even though inflammatory conditions are known to exert adverse effects on bone metabolism, there are no published data regarding SARS-CoV-2 infection and subsequent fracture risk. We present a brief review of the molecular mechanisms linking inflammatory diseases to increased fracture risk/osteoporosis and of the therapeutic strategies that can prevent bone resorption in patients with inflammatory disease, focusing on the RANK-RANKL system. We also make some considerations on gender differences in infection response and on their implications for survival and for the consequences of COVID-19. Several inflammatory cytokines, especially IL-1, IL-6, and TNF-α, stimulate osteoclast activity, favoring bone resorption through the RANK-RANKL system. Data from the previous SARS-CoV outbreak suggest that the present disease also has the potential to act directly on bone resorption units, although confirmation is clearly needed. Even though the available data are limited, the RANK-RANKL system may provide the best therapeutic target to prevent bone resorption after COVID-19 disease. Vitamin D supplementation in case of deficiency could definitely be beneficial for bone metabolism, as well as for the immune system. Supplementation of vitamin D in case of deficiency could be further advantageous. In COVID-19 patients, it would be useful to measure the bone metabolism markers and vitamin D. Targeting the RANK-RANKL system should be a priority, and denosumab could represent a safe and effective choice. In the near future, every effort should be made to investigate the fracture risk after SARS-CoV-2 infection.

## Introduction

In early December 2019, a novel coronavirus emerged in Wuhan City (Hubei Province, China). It subsequently spread throughout the country and eventually through the world. The virus, called severe acute respiratory syndrome coronavirus 2 (SARS-CoV-2), can easily reach the lower respiratory tract, causing a bilateral pneumonia (coronavirus infectious disease, COVID-19) characterized by a high mortality rate [1]. According to the information reported in the World Health Organization website, by May 18, the number of laboratory-confirmed cases of COVID-19 worldwide had climbed to 4,660,870 and 313,403 patients had died. In Italy, on the same day, the number of laboratory-confirmed COVID-19 cases was 226,699; average patient age was 62 years (52.1% men, 47.9% women).

As SARS-CoV-2 infection predominantly affects older people [2], one of the aspects to be evaluated in the near future is its interaction with bone metabolism/osteoporosis. Osteoporosis, a systemic disease of the skeleton, is characterized by a progressive quantitative and qualitative alteration of the bone mass, which is accompanied by an increase in the risk of fracture even in the absence of trauma [3, 4]. In Italy, osteoporosis is the third most common chronic disease (after hypertension and osteoarthritis/arthritis) and affects about 3.5 million women and 1 million men [5]. The annual incidence of femoral fracture has been estimated at 87,000 cases, whereas the overall prevalence of vertebral fractures has been estimated at 155,000 a year. Altogether, 465,000 new fractures occur in Italy every year [6].

So far, data on bone metabolism in SARS-COV-2 infection are very scanty. Moreover, it is unclear whether the incidence of osteoporosis and the risk of fracture may increase in patients after recovery from the infection, due to the interaction of their risk factors (e.g., old age, smoking habits, long-term bed confinement, hypovitaminosis D, and steroid treatment) with the COVID-19 inflammatory process, even though according to a recent report fracture patients may be at higher risk of COVID-19 [7].

## Immunology Considerations

According to the U.S. Center for Disease Control (CDC) guidelines revised on June 20, 2020, the signs and symptoms of COVID-19 present at illness onset vary but include the following: fever or chills, cough, shortness of breath or difficulty breathing, fatigue, muscle or body aches, headache, new loss of taste or smell, sore throat, congestion or runny nose, nausea and vomiting, diarrhea, and acute respiratory distress syndrome [8]. According to China’s National Health Commission Guidelines for diagnosis and management of COVID-19, severe cases of COVID-19 have been defined by percutaneous oxygen saturation (spO_2_) ≤ 93% or respiratory rates ≥ 30/min [9].

Zhang et al. [10] have found that disease severity correlates directly with blood leukocyte and neutrophil count, neutrophil percentage. In contrast, other researchers have described leukopenia, rather than leukocytosis, as a common finding in patients with severe COVID-19 disease [11–13]. The markedly higher serum concentrations of pro- and antiinflammatory cytokines, including IL-6, TNF-α, and IL-10, characterizing severe compared with moderate cases, suggest that disease severity could be associated to a “cytokine storm” [14].

In agreement with this hypothesis, T lymphocytes are considered as the main target cells in SARS due to COVID-19, triggering the cytokine storm with subsequent exhaustion of the immune response. Interestingly, no association between viral load and disease severity has been reported [15]. A large study of 522 patients with laboratory-confirmed COVID-19 has highlighted a dramatic reduction of total T cells, CD4+, and CD8+ T cells, especially among patients requiring intensive care unit care, suggesting that cytokine hypersecretion during COVID-19 infection does not originate from T cells but from monocytes and macrophages [16], which are also osteoclast precursors. However, irrespective of the cytokine source, it is now established that the immune system amplifies and maintains the cytokine storm triggered by contact with SARS-CoV-2.

Given the potential role of cortisone in modulating the inflammatory response, the use of steroid therapy to stop the cytokine cascade has been studied. Although initial data suggested using these drugs with great caution because of the possible risk of worsening lung injury from COVID-19 [17], recent preliminary data results from the RECOVERY trial has shown that low dose dexamethasone (6 mg once daily) can reduce by 8–26% mortality in patients with severe COVD-19 [18]. By the way, it is logical to expect a negative effect of this treatment on bone health, given that both single boluses of high-dose cortisone and prolonged treatments with low-dose steroids can suppress both bone formation and reabsorption, with a net loss of bone mineral mass [19].

It is conceivable that bone complications related to infection or treatment will emerge in the next few months as they did after the SARS outbreak of 2002–2003. At the time, reports of arthralgia, reduced mineral bone density (BMD), and osteonecrosis of femurs and tibiae could only partially be explained by high-dose steroid treatment. A subsequent in vitro study showed that a specific SARS-CoV protein, 3a/X1, directly promotes osteoclastogenesis, accelerating osteoclast differentiation from monocyte/macrophage precursors, enhancing the expression of receptor activator of NF-kB ligand (RANKL) and inflammatory cytokines such as TNF-α, which indirectly promote osteoclastogenesis [20].

## Vitamin D Deficiency

From an epidemiological point of view, it is interesting to note that mortality from COVID-19 between different countries shows a latitude-dependent variability which largely overlaps the prevalence of hypovitaminosis D in the world [21].

In subjects suffering from hypovitaminosis D, observational studies have shown a higher prevalence of cardiovascular complications (e.g., myocardial infarction or ischemic stroke) [22]; low vitamin D levels are present in many endocrine diseases as hyperaldosteronism [23]; but there are also numerous evidences to support a direct role of vitamin D in modulating the immune response. Epidemiologic studies have shown that vitamin D insufficiency is associated with respiratory infections in children, newborns, pregnant women, and adults [24].

During microbial infection, macrophages are activated into M1 and M2 macrophages. M1 macrophages produce proinflammatory mediators (e.g., IL-6 and TNF-α) whereas M2 macrophages produce the antiinflammatory molecule IL-10. In vitro, calcitriol stimulates the differentiation from monocytes to macrophages and, at the same time, decreases the production of proinflammatory factors by activated macrophages. Moreover, calcitriol directly regulates the expression of several antimicrobial peptides by innate immune cells (e.g., CAMP and β-defensins) [25]. Taken together, these data suggest that vitamin D may enhance the initial response to infection by increasing the efficiency of the innate response, subsequently protecting the organism from excessive cytokine activation. Regarding COVID-19, the interaction of the SARS-CoV-2 spike glycoprotein with the human dipeptidyl peptidase-4 receptor (DPP-4/CD26) could be an important virulence factor. DPP-4/CD26 receptor expression can be reduced in vivo by correction of hypovitaminosis D, further suggesting that optimization of vitamin D status could improve the outcome COVID-19 patients [26]. Unfortunately, most of the immunomodulatory effects of vitamin D become evident only when serum concentrations of 25(OH)D reach 40–80 ng/ml, which can be obtained, in vivo, thanks to the local hydroxylation of vitamin D by cells of the immune system (macrophages, lymphocytes, and dendritic cells) [25].

At present, large population studies are needed to be able to draw definitive conclusions on the relationship between hypovitaminosis D and susceptibility to COVD-19 infection. On this purpose, a recent, large, epidemiological study was conducted on over 500,000 participants aged 37–73 years from the UK to establish the association between ethnicity, vitamin D levels and susceptibility to COVID-19 infection. Authors have initially suggested an association between COVID-19 infection and vitamin D, since they demonstrated a higher risk of confirmed COVD-19 infection in ethnic minority groups with lower 25(OH)D concentration, but this result was not confirmed after adjustment for confounders, and further evidence is still needed [27].

## Gender Considerations

Most reports indicate that males are more susceptible to SARS-CoV-2 infection than females, and that older men with underlying comorbidities are more likely to develop severe COVID-19 disease 9–11. As in the case of the SARS-CoV outbreak of 2002–2003, gender seems to be a risk factor for higher severity and mortality in patients with COVID-19 independently of age and susceptibility [28].

Even though the influence of a different exposure to risk factors (e.g., smoking habits or alcohol intake) cannot be totally ruled out, it is possible that this difference is related to gender-specific genetic and hormonal factors. In fact, data from epidemiological studies have revealed higher mortality rates from various infectious diseases in men than in females, whereas women are more susceptible to autoimmune diseases.

The biological basis for this discrepancy lies partially in their different innate immune response: the activity of innate immune cells (i.e., monocytes, macrophages and denditric cells) is modulated by sexual hormones (e.g., estrogen reduces IL-6 expression and delays the spontaneous apoptosis of neutrophils, whereas the testosterone has immunosuppressive effects, dampening lymphocyte proliferation), and the X chromosome encodes many innate immune molecules [29]. As for COVID-19, previous studies of surgical series have shown that the male gender is an independent risk factor for the development of major infections and for septic shock [30]. In animal models, after experimentally induced injury, females show lower levels of IL-6 and IL-10 and enhanced survival, suggesting that an unbalanced production of proinflammatory mediators could explain the unfavorable outcomes of males [29]. Interestingly, as noted above, estrogen reduces the expression of IL-6, which is one of the main targets of COVID-19 treatment. As will be explained later in the text, IL-6 represents an important cofactor for bone resorption in inflammatory diseases; therefore, during SARS-CoV-2 infection, men, though less affected by osteoporosis, may experience more bone metabolism alterations than women for higher levels of IL-6 resulting from the lack of suppression by estrogen.

## Osteoimmunology

The interaction between inflammatory molecules and the bone system is defined as “osteoimmunology” [31]. In this context, receptor activator of NF-kB (RANK) and RANKL, though initially identified as mediators of T cell activation, were subsequently found to play a pivotal role in osteoclastogenesis in physiological and pathological conditions.

**Fig. 1:**
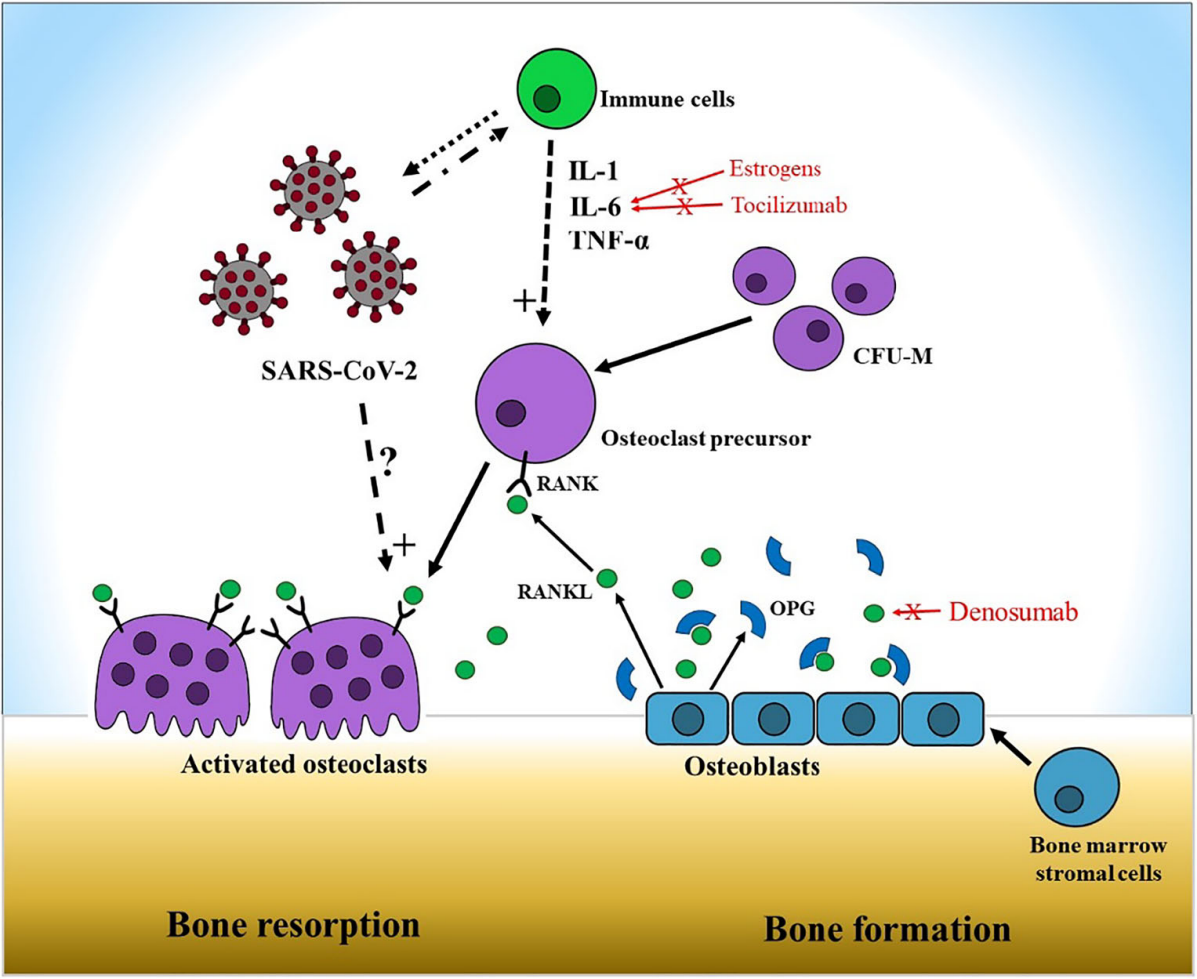
The RANK-RANKL-OPG system. Bone metabolism is the result of the balance between bone resorption by osteoclasts and bone formation by osteoblasts. RANK is a transmembrane protein expressed by osteoclast precursors that derive from the colony-forming unit for macrophages (CFU-M). The binding of RANKL, expressed by osteoblasts, stimulates the formation and the activity of the osteoclasts, favoring bone resorption. OPG, an additional soluble protein produced by osteoblasts, prevents the binding between RANK and RANKL, counterbalancing bone resorption. Immune cells such as activated T cells and inflammatory cytokines act synergistically promoting the RANK-RANKL pathway. SARS-Cov-2 could have an additive effect promoting the cytokine storm which promotes bone resorption, but a direct effect of the virus on osteoclastic activation cannot be excluded. From a pharmacological point of view, estrogens and tocilizumab can reduce inflammatory cytokine levels (specially IL-6), whereas denosumab directly inhibits the RANK-RANKL interaction and they all represent potential protective agents against bone resorption

RANKL is a transmembrane protein mainly expressed by osteoblasts, periosteal cells, and osteocytes, whereas its receptor, RANK, is expressed by osteoclasts and preosteoclasts. After binding to RANK, RANKL stimulates the formation and activity of osteoclasts, which are responsible for bone resorption. However, osteoblasts produce an additional soluble protein, osteoprotegerin (OPG), which acts as a “decoy receptor”, preventing the binding of RANKL to RANK and, consequently, osteoclast activation. Under physiological conditions, the ratio of RANKL to OPG is balanced, and bone resorption is counterbalanced by bone deposition. Aging, estrogen deficiency, and systemic glucocorticoid exposure can alter this ratio in favor of RANKL, causing a progressive reduction of BMD that may result in the clinical condition of osteoporosis [32]. The RANK-RANKL system has been extensively studied in the context of osteoporosis related to chronic inflammatory disease. In fact, rheumatic diseases such as rheumatoid arthritis (RA) and ankylosing spondylitis are frequently associated with bone loss, both localized and systemic, and are characterized by an increased risk of osteoporotic fractures in all age groups [33] (Fig. 1).

**Fig. 2:**
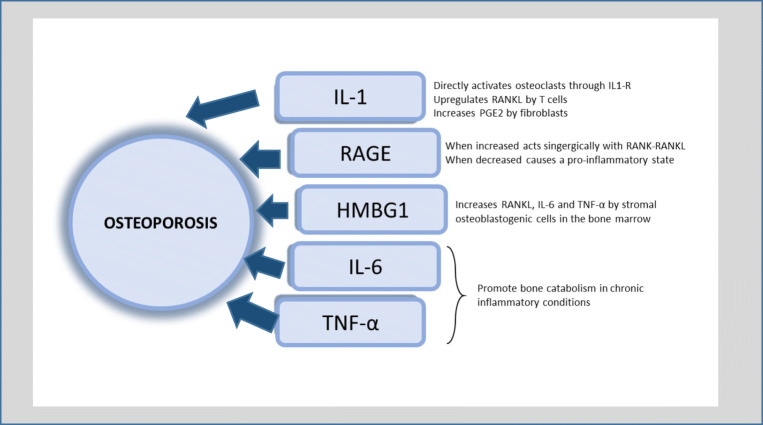
Proposed mechanism of alarmins. Modified from Fig. 1 in De Martinis et al. [22]

In a recent review, De Martinis et al. [34] have summarized the main mechanism involved in the activation of osteoclastogenesis by inflammatory phenomena. They stressed that infection, trauma, and injuries induce the production of endogenous signaling mediators of inflammatory response named “alarmins”. Alarmins are released by mesenchymal cells and act as chemotactic factors and pattern recognition receptors, allowing the innate immune cells to be alerted to tissue damage. By recalling the cells of the immune system, these molecules initiate tissue repair processes but, at the same time, are also responsible for the bone resorption that occurs during acute inflammatory phenomena. Among the most important molecules involved, the authors mention IL-1, receptor for advanced glycation end products (RAGE), and high mobility group box 1 protein (HMGB1) protein (Fig. 2).

IL-1 is a proinflammatory cytokine, also known as “osteoclast activating factor”, that increases osteoclast vitality and resorptive activity by an independent and RANKL-dependent mechanism. In fact, osteoclasts express on their surface the specific IL-1 receptor (IL1-R), whose expression is further increased by RANKL, which directly promotes their resorptive activity through the same intracellular pathway, RANK-RANKL. Moreover, as in the case of other inflammatory cytokines such as IL-18 and TNF-α, IL-1 upregulates RANKL production by T cells and increases PGE2 synthesis in fibroblasts, with a consequent additional activation of osteoclastogenesis. RAGE is a multiligand receptor belonging to the immunoglobulin receptor superfamily, whose circulating levels correlate with the risk of osteoporosis. Higher levels of RAGE are common in chronic conditions such as diabetes, cancer, cardiovascular disease, and neurodegeneration. RAGE is expressed by mesenchymal cells (osteocytes, osteoblasts, and osteoclasts) and guides their growth and development. It binds endogenous factors, like advanced glycation end products (AGE) and HMGB1 protein, which are released in case of stress or cell death, and activates the downstream signaling pathways (AP-1, NFAT, NF-κB, STAT3, and CREB) involved in osteoclastogenesis, acting synergistically with the RANK-RANKL system. Notably, even reduced levels of RAGE have a negative effect on bone metabolism: it has been suggested that its overexpression in inflammatory conditions could have a role in inflammation resolution and tissue repair, whereas insufficient RAGE expression induces suppression of PPAR-α and its cofactor PGC1-α, leading to a proinflammatory state and to BMD decrease. Finally, HMGB1, a nuclear protein secreted by macrophages after RANKL binding, is required for osteoclast formation and TNF-α expression. It acts as an alarmin in several tissues, and it binds RAGE and toll-like receptors (TLR) of immune cells, amplifying the inflammatory process. At the bone level, HMGB1 increases the expression of RANKL, IL-6, and TNF-α by stromal osteoblastogenic cells of the bone marrow and acts as a chemotactic stimulus for mature osteoclasts [28]. Moreover, RANKL is directly expressed by CD4+ T cells, which activate osteoclasts and stimulate other RANKL-expressing cells such as monocytes/macrophages, promoting bone catabolism [35]^.^

Inflammatory molecules are also physiologically produced during aging, in a process called “inflammaging” (inflammation plus aging). Circulating levels of TNF-α, IL-6, and CRP progressively increase with aging and are overexpressed in chronic conditions such as atherosclerosis, diabetes mellitus, and chronic obstructive pulmonary disease. As in the case of rheumatic diseases, elderly subjects with chronic disease show a higher prevalence of osteoporosis. In vitro studies have suggested that IL-1, IL-6, and TNF-α could have a role in reducing BMD in these patients, although the in vivo data are conflicting. The recent OsteoLaus study has found no correlations between circulating inflammatory molecules and bone metabolism parameters in postmenopausal women after exclusion of acute inflammatory conditions [36].

The discovery of the RANK-RANKL pathway has led to the development of denosumab, a fully human antibody that binds to and inhibits RANKL, thus preventing bone resorption, which has been shown to reduce the risk of vertebral, nonvertebral, and hip fractures in postmenopausal women with osteoporosis [37]. In patients with RA, denosumab given subcutaneously for 6–12 months inhibited the occurrence and progression of bone erosions [38, 39], even though this effect raised doubts about a possible immunosuppressive effect.

These considerations suggest that during the current pandemic, denosumab could be the best therapeutic choice to preserve bone from the adverse effect of SARS-CoV-2 infection, correcting the RANK-RANKL system imbalance due to the massive cytokine release and to high-dose steroid treatment.

Currently, the mainstay of COVID-19 pharmacological treatment is represented by antiviral drugs, whose action is directed toward structural elements of the virus, and by immune system modulators such as glucocorticoids and antirheumatic drugs, which dampen the excess of cytokines produced in response to disease [40]. Interestingly, a side effect of antirheumatic drugs could be bone loss prevention. In RA patients, hydroxychloroquine has been shown to prevent bone resorption (measured by β-CTx levels) after 3 and 6 months of treatment [33]. Above all, tocilizumab, a monoclonal antibody that binds to IL-6 receptor and which is currently one of the most promising drugs for COVID-19 treatment [40], has long been approved for use in RA patients, where it reduces bone turnover and improves BMD [33].

## Conclusions

In conclusion, bone metabolism and inflammation are linked by several biological and clinical mechanisms. In COVID-19 patients, it would be useful to measure the bone metabolism markers (CTX, P1NP, alkaline phosphatase). Targeting the RANK-RANKL system should be a priority, and denosumab could represent a safe and effective choice. At present, there are no studies evaluating the contemporary association between tocilizumab and denosumab: retrospective analysis of patients who had received denosumab before infection and who underwent tocilizumab therapy could provide information about this. Vitamin D deficiency should be sought in all patients, and vitamin D supplementation could be helpful for positive influence on the immune system and bone metabolism. In the near future, every effort should be made to investigate the fracture risk in patients who have recovered from SARS-CoV-2 infection, both females and also males.

## References

[1] Pedersen SF, Ho Y-C (2020). SARS-CoV-2: a storm is raging. J Clin Invest.

[2] Zhou F, Yu T, Du R, Fan G, Liu Y, Liu Z, Hiang J, Wang J, Song B, Gu X, Guan L, Wei Y, Li H, Wu X, Xu J, Tu S, Zhang Y, Chen H, Cao B (2020). Clinical course and risk factors for mortality of adult inpatients with COVID-19 in Wuhan, China: a retrospective cohort study. Lancet..

[3] Rossini M, Adami S, Bertoldo F, Diacinti D, Gatti D, Giannini S, Giusti A, Malavolta N, Minisola S, Osella G, Pedrazzoni M, Sinigaglia L, Viapiana O, Isaia GC (2016). Guidelines for the diagnosis, prevention and management of osteoporosis. Reumatismo..

[4] Nuti R, Commissione Intersocietaria per l’Osteoporosi SIE, SIMFER, SIMI, SIOT, SIGG, SIMG, SIOMMMS S. Linee Guida sulla gestione dell’Osteoporosi e delle Fratture da fragilità. 2017. https://www.sigg.it/wp-content/uploads/2018/05/Linee-Guida-definitive-OSTEOPOROSI-1.pdf. Accessed 11 Apr 2020.

[5] Hernlund E, Svedbom A, Ivergård M, Compston J, Cooper C, Stenmark J, McCloskey EV, Jönsson G, Kanis JA (2013). Osteoporosis in the European Union: medical management, epidemiology and economic burden: a report prepared in collaboration with the International Osteoporosis Foundation (IOF) and the European Federation of Pharmaceutical Industry Associations (EFPIA). Arch Osteoporos.

[6] Rossini M. Epidemiology and economic burden of osteoporosis in Italy | IRIS Verona. 2013. https://iris.univr.it/handle/11562/626966#.XpGYIsgzaUk.

[7] Mi B, Chen L, Xiong Y, Xue H, Zhou W, Liu G (2020). Characteristics and early prognosis of COVID-19 infection in fracture patients. J Bone Jt Surg Am.

[8] Interim clinical guidance for management of patients with confirmed Coronavirus Disease (COVID-19). Centers for Disease Control and Prevention. 2020. https://www.cdc.gov/coronavirus/2019-ncov/hcp/clinical-guidance-management-patients.html. Accessed 02 Aug 2020.

[9] Novel Coronavirus Treatment Guidelines – 7th Edition (English Translation on ELotus.com). China's National Health Commission. 2020. https://www.elotus.org/promo-files/COVID-19_resources/Guidance%20for%20Corona%20Virus%20Disease%202019%20(English%207th%20Edition%20Draft).pdf. Accessed 02 Aug 2020.

[10] Zhang G, Wang B. Analysis of clinical characteristics and laboratory findings of 95 cases of 2019 novel coronavirus pneumonia in Wuhan China : a retrospective analysis. Respir Res. 2019. 10.21203/rs.3.rs-17712/v1. 10.1186/s12931-020-01338-8 PMC7099829 32216803

[11] Huang C, Wang Y, Li X, Ren L, Zhao J, Hu Y, Zhang L, Fan G, Xu J, Gu X, Cheng Z, Yu T, Xia J, Wei Y, Wu W, Xie X, Yin W, Li H, Liu M, Xiao Y, Gao H, Guo L, Xie J, Wang G, Jiang R, Gao Z, Jin Q, Wang J, Cao B (2020). Clinical features of patients infected with 2019 novel coronavirus in Wuhan. China Lancet.

[12] Guan WJ, Ni ZY, Hu Y, Liang WH, Ou CQ, He JX, et al. China Medical Treatment Expert Group for Covid-19. Clinical characteristics of Coronavirus Disease 2019 in China. N Engl J Med. 2020. 10.1056/NEJMoa2002032.

[13] Chen N, Zhou M, Dong X, Qu J, Gong F, Han Y, et al. Epidemiological and clinical characteristics of 99 cases of 2019 novel coronavirus pneumonia in Wuhan, China: a descriptive study. Lancet. 2020. 10.1016/S0140-6736(20)30211-7. 10.1016/S0140-6736(20)30211-7 PMC7135076 32007143

[14] Chen G, Wu D, Guo W, Cao Y, Huang D, Wang H, Wang T, Zhang X, Chen H, Yu H, Zhang X, Zhang M, Wu S, Song J, Chen T, Han M, Li S, Luo X, Zhao J, Ning Q (2020). Clinical and immunologic features in severe and moderate forms of coronavirus disease 2019. J Clin Invest.

[15] Georgiev T (2020). Coronavirus disease 2019 (COVID-19) and anti-rheumatic drugs. Rheumatol Int.

[16] Diao B, Wang C, Tan Y, Chen X, Liu Y, Ning L, et al. Reduction and functional exhaustion of T cells in patients with coronavirus disease 2019 (COVID-19). Front Immunol. 2020. 10.1101/2020.02.18.20024364. 10.3389/fimmu.2020.00827 PMC7205903 32425950

[17] Russell CD, Millar JE, Baillie JK (2020). Clinical evidence does not support corticosteroid treatment for 2019-nCoV lung injury. Lancet..

[18] Mahase E (2020). Covid-19: Low dose steroid cuts death in ventilated patients by one third, trial finds. BMJ.

[19] Ton FN, Gunawardene SC, Lee H, Neer RM (2005). Effects of low-dose prednisone on bone metabolism. J Bone Miner Res.

[20] Obitsu S, Ahmed N, Nishitsuji H, Hasegawa A, Nakahama K, Morita I, Nishigaki K, Hayashi T, Masuda T, Kannagi M (2009). Potential enhancement of osteoclastogenesis by severe acute respiratory syndrome coronavirus 3a/X1 protein. Arch Virol.

[21] Braiman M. Latitude dependence of the COVID-19 mortality rate—a possible relationship to vitamin D deficiency? SSRN. 2020; 10.2139/ssrn.3561958

[22] Muscogiuri G, Annweiler C, Duval G, Karras S, Tirabassi G, Salvio G, Balercia G, Kimball S, Kotsa K, Mascitelli L, Bhattoa HP, Colao A (2017). Vitamin D and cardiovascular disease: from atherosclerosis to myocardial infarction and stroke. Int J Cardiol.

[23] Ceccoli L, Ronconi V, Giovannini L, Marcheggiani M, Turchi F, Boscaro M, Giacchetti G (2013). Bone health and aldosterone excess. Osteoporos Int.

[24] Colotta F, Jansson B, Bonelli F (2017). Modulation of inflammatory and immune responses by vitamin D. J Autoimmun.

[25] Vanherwegen AS, Gysemans C, Mathieu C (2017). Regulation of immune function by vitamin D and its use in diseases of immunity. Endocrinol Metab Clin N Am.

[26] McCartney DM (2020). Byrne DG optimisation of vitamin D status for enhanced Immuno-protection against Covid-19. Ir Med J.

[27] Hastie CE, Mackay DF, Ho F, Celis-Morales CA, Katikireddi SV, Niedzwiedz CL, Jani BD, Welsh P, Mair FS, Gray SR, O’Donnell CA, Gill JMR, Sattar N, Pell JP (2020). Vitamin D concentrations and COVID-19 infection in UK biobank. Diabetes Metab Syndr.

[28] Jin JM, Bai P, He W, Wu F, Liu XF, Han DM, et al. Gender differences in patients with COVID-19: Focus on severity and mortality. medRxiv. 2020. 10.1101/2020.02.23.20026864. 10.3389/fpubh.2020.00152 PMC7201103 32411652

[29] Jaillon S, Berthenet K, Garlanda C (2019). Sexual dimorphism in innate immunity. Clin Rev Allergy Immunol.

[30] Offner PJ, Moore EE, Biffl WL (1999). Male gender is a risk factor for major infections after surgery. Arch Surg.

[31] Ralston SH, Schett G (2018). Osteoimmunology. Calcif Tissue Int.

[32] Sinningen K, Tsourdi E, Rauner M, Rachner TD, Hamann C, Hofbauer LC (2012). Skeletal and extraskeletal actions of denosumab. Endocrine..

[33] Dubrovsky AM, Lim MJ, Lane NE (2018). Osteoporosis in rheumatic diseases: anti-rheumatic drugs and the skeleton. Calcif Tissue Int.

[34] De Martinis M, Ginaldi L, Sirufo MM, Pioggia G, Calapai G, Gamgemi S, et al. Alarmins in osteoporosis, RAGE, IL-1, and IL-33 pathways: a literature review. Medicina (Kaunas). 2020;56(3). 10.3390/medicina56030138. 10.3390/medicina56030138 PMC7142770 32204562

[35] Kong YY, Feige U, Sarosi I, Bolon B, Tafuri A, Morony S, Capparelli C, Li J, Elliott R, McCabe S, Wong T, Campagnuolo G, Moran E, Bogoch ER, Van G, Nguyen LT, Ohashi PS, Lacey DL, Fish E, Boyle WJ, Penninger JM (1999). Activated T cells regulate bone loss and joint destruction in adjuvant arthritis through osteoprotegerin ligand. Nature..

[36] Fischer J, Hans D, Lamy O, Marques-Vidal P, Vollenweider P, Aubry-Rozier B. “Inflammaging” and bone in the OsteoLaus cohort. Immun Ageing. 2020. 10.1186/s12979-020-00177-x. 10.1186/s12979-020-00177-x PMC7057650 32158491

[37] Faienza MF, Chiarito M, D'amato G (2018). Monoclonal antibodies for treating osteoporosis. Expert Opin Biol Ther.

[38] Geusens P (2012). The role of RANK ligand/osteoprotegerin in rheumatoid arthritis. Ther Adv Musculoskelet Dis.

[39] Tanaka Y, Ohira T (2018). Mechanisms and therapeutic targets for bone damage in rheumatoid arthritis, in particular the RANK-RANKL system. Curr Opin Pharmacol.

[40] Barlow A, Landolf KM, Barlow B, Yeung SYA, Heavner JJ, Claassen CW, Heavner MS (2020). Review of emerging pharmacotherapy for the treatment of coronavirus disease 2019. Pharmacother J Hum Pharmacol Drug Ther.

